# CAR-T Plus Radiotherapy: A Promising Combination for Immunosuppressive Tumors

**DOI:** 10.3389/fimmu.2021.813832

**Published:** 2022-01-12

**Authors:** Vicky Mengfei Qin, Nicole M. Haynes, Criselle D’Souza, Paul J. Neeson, Joe Jiang Zhu

**Affiliations:** ^1^Cancer Immunology Program, Peter MacCallum Cancer Centre, Melbourne, VIC, Australia; ^2^Department of Clinical Pathology, Faculty of Medicine, Dentistry and Health Sciences, University of Melbourne, Melbourne, VIC, Australia; ^3^Division of Cancer Research, Peter MacCallum Cancer Centre, Melbourne, VIC, Australia; ^4^Sir Peter MacCallum Department of Oncology, Faculty of Medicine, Dentistry and Health Sciences, University of Melbourne, Melbourne, VIC, Australia

**Keywords:** radiotherapy (RT), chimeric antigen receptor T cell (CAR-T), solid tumor, immunosuppression, tumor microenvironment (TME)

## Abstract

Radiotherapy (RT) is the standard-of-care treatment for more than half of cancer patients with localized tumors and is also used as palliative care to facilitate symptom relief in metastatic cancers. In addition, RT can alter the immunosuppressive tumor microenvironment (TME) of solid tumors to augment the anti-tumor immune response of immune checkpoint blockade (ICB). The rationale of this combination therapy can also be extended to other forms of immunotherapy, such as chimeric antigen receptor T cell (CAR-T) therapy. Similar to ICB, the efficacy of CAR-T therapy is also significantly impacted by the immunosuppressive TME, leading to compromised T cell function and/or insufficient T cell infiltration. In this review, we will discuss some of the key barriers to the activity of CAR-T cells in the immunosuppressive TME and focus on how RT can be used to eliminate or bypass these barriers. We will present the challenges to achieving success with this therapeutic partnership. Looking forward, we will also provide strategies currently being investigated to ensure the success of this combination strategy in the clinic.

## Introduction

Adoptive cell transfer (ACT) has shifted the therapeutic paradigm for cancer patients in recent years. Transducing T cells with a chimeric antigen receptor (CAR) to redirect their antigen specificity against a defined tumor antigen has further broadened the use of ACT. CAR-T cells can recognize tumor-associated surface antigens *via* the single-chain variable fragment (scFv) and initiate anti-tumor immune responses by intracellular signaling domains, such as CD3ζ and CD28 (1). CAR-T therapy has demonstrated remarkable anti-cancer activity, achieving long-term remission in patients with refractory B cell malignancies (2). However, similar success with CAR-T cell therapy has not been achieved in solid tumors (3).

Solid tumors can be characterized into two distinct subsets based on the inflammatory status of the TME (4). Tumors (eg. melanoma) with a high inflammation signature tend to respond well to ICB (5), an effect largely mediated by CD8^+^ effector T cells (6, 7); however, the development of resistance to immunotherapy is common. The presence of liver metastases is also being increasingly recognized as a barrier to ICB efficacy, even in the context of melanoma (8, 9). Tumors that support an immune excluded or deserted TMEs, such as prostate and pancreatic cancers, are described as immunosuppressive (10, 11), and typically fail to respond to ICB and CAR-T therapy. These immunosuppressive tumors lack T-cell chemokines to drive the recruitment of CD8^+^ effector T cells or CAR-T cells and are also enriched with suppressor cells that compromise T cell persistence and function. To overcome these immunosuppressive features, the value of TME-altering therapies, such as RT, is actively being explored.

**Figure 1:**
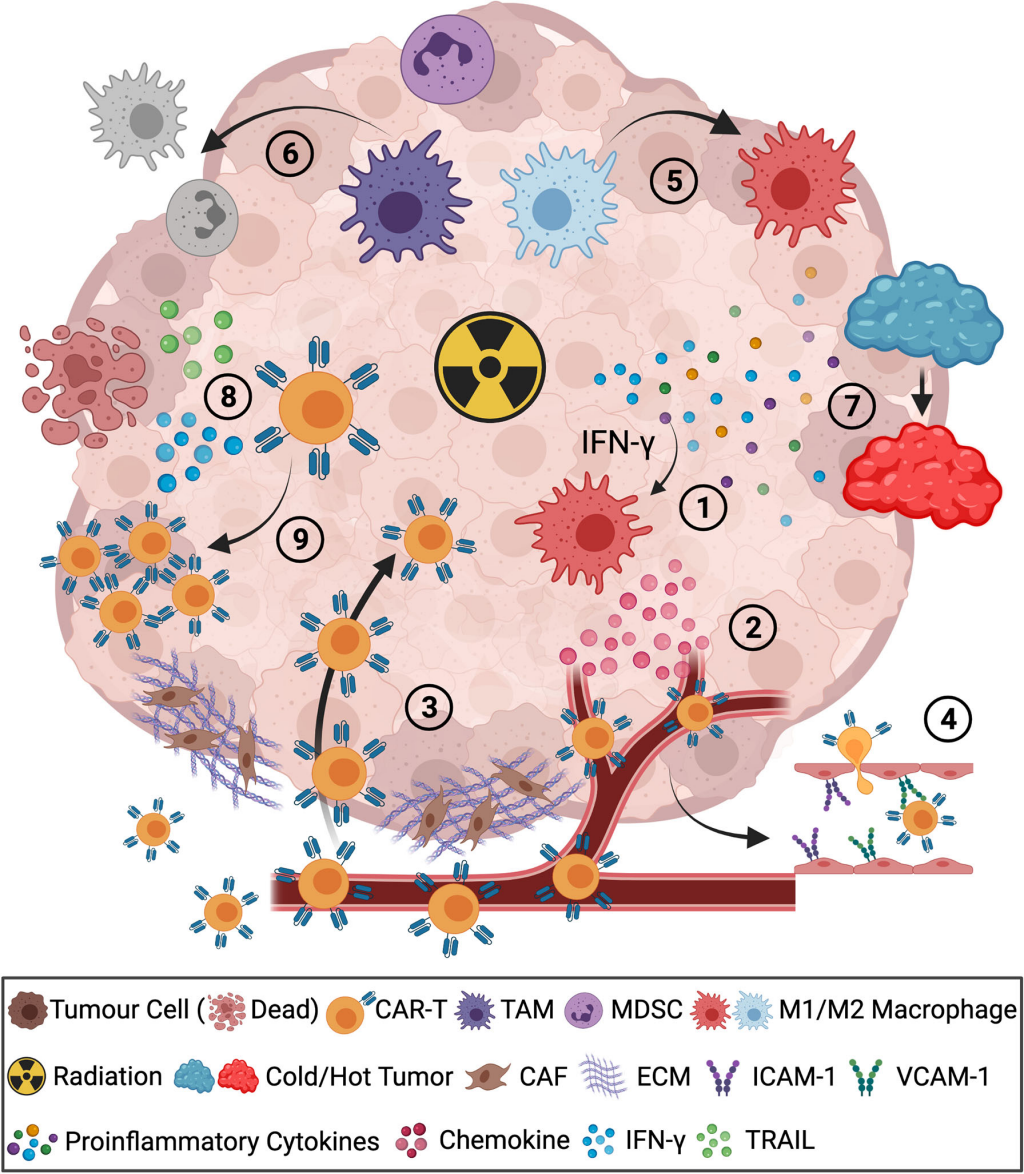
Radiotherapy improves the outcomes of CAR-T cells in combination therapy. ①Radiation-induced IFN-γ promotes chemokine secretion of CXCL9/10/11, ②leading to effective CAR-T cell homing to the tumor bed. ③Diminished tumor barriers of cancer-associated fibroblasts (CAF) and extracellular matrix (ECM) promote CAR-T cell infiltration. ④Radiation-induced expression of ICAM-1/VCAM-1 on the endothelium of tumor vasculature facilitated CAR-T cell infiltration. ⑤RT polarized M2 macrophages to M1 macrophages in the TME. ⑥Reduction of TAM and MDSC by RT. ⑦Radiation-induced increased expression of proinflammatory cytokines altered the TME from immunosuppressive “cold” to immune-inflamed “hot”. ⑧Radiation enhanced infiltrated CAR-T cell function with increased expression of TRAIL, IFN-γ and ⑨augmented expansion of CAR-T cells. The figure is created with BioRender.com.

RT is the standard-of-care treatment used for curative or palliative intent in close to 50% of cancer patients (12). Factors including total dose, fractionation scheme, hypoxia, and the intrinsic radiosensitivity of tumor cells all come into play in influencing the overall impact of RT on the TME (13–15) and its ability to reactivate host anti-tumor immune responses (16). Evidence to support the rationale for combining CAR-T cells with radiation therapy is growing. The RT effect on the TME, and the mechanisms whereby this occurs, have been widely studied in a variety of tumor models using different dose/fractionation schemes (**Table 1**). As a TME-modifying therapy, RT can induce the release of chemokines, such as CXCL9, to augment T cell trafficking (**Figure 1** ①/②), increase the expression of adhesion molecules that may promote T cell infiltration (**Figure 1** ③/④), alter the immune cell composition in the TME (**Figure 1** ⑤/⑥), and increase the expression of immune-stimulatory cytokines to enhance the functional activity of effector T cells in the TME (**Figure 1** ⑧/⑨) (32). By changing the TME and creating a niche for immune cells, these benefits may synergize with immunotherapies, including CAR-T therapy.

**Table 1:** Evidence of the potential synergistic effect of RT in combination with CAR-T cells.

Tumor model	Scheme	Mechanism	Reference
**Preclinical evidence of RT as a TME altering therapy**			
Melanoma	1 × 15Gy	RT-induced type I IFN promoted CXCL10 expression, leading to increased CD8^+^ T cell infiltration.	Lim et al (17),
Multiple models	3 × 8Gy	RT activated the STING pathway and induced type I IFNs to recruit DC and CD8^+^ T cells.	Vanpouille-Box et. al (18),
Prostate cancer	10 × 2Gy	RT remodeled the tumor vasculature and improved oxygenation.	Potiron et al. (19),
Non-small cell lung carcinoma	1 × 18Gy and 3 × 6Gy	Irradiated CAF decreased the pro-tumorigenic potential that affected angiogenesis and tumor growth.	Grinde et al. (20),
Breast cancer	3 × 8Gy	RT induced up-regulation of ICAM-1 to enhance both the activation and tumor infiltration of CD8^+^ T cells.	Zhao et al. (21),
Melanoma	1 × 15Gy	RT-induced IFN-γ increased the VCAM-1 expression on tumor vasculature to facilitate T cell infiltration.	Lugade et al. (22),
Multiple models	1 × 6Gy	Liver-directed RT eliminated immunosuppressive hepatic macrophages and increased T cell function in liver metastatic models.	Yu et al. (23),
Lung adenocarcinoma	2 × 1Gy	RT induced M1 macrophage polarization and enhanced immune cell infiltration.	Barsoumian et al. (24),
Multiple models	2 × 12.5Gy	RT downregulated the expression of VEGF to reduce the recruitment of MDSC into tumors.	Lan et al. (25),
Prostate cancer	2 × 10Gy	RT induced CXCL9 expression, leading to increased CD8^+^ T cell infiltration.	Keam et al. (26),
**Preclinical evidence of RT in combination with CAR-T cells**			
Glioblastoma	1 × 5Gy	RT facilitated vasculature normalization to promote CAR-T cell extravasation in the TME.	Murty et al. 2020 (27)
Pancreatic cancer	1 × 2Gy	RT sensitized antigen-negative tumor cells to TRAIL-dependent killing mediated by CAR-T cells.	DeSelm et al. 2018 (28)
Glioblastoma	1 × 4Gy	RT boosted CAR-T cell activity (IFN-γ production) and upregulated CAR-targeted stress ligand.	Weiss et al. 2018 (29)
**Clinical studies of RT in combination with CAR-T cells**			
Diffuse large B-cell lymphoma	20 × 2Gy	RT was related to CAR-T cell expansion and therapeutic durability of CAR-T cell therapy.	NCT03196830 (30)
Multiple Myeloma	5 × 4Gy	The synergistic abscopal effect induced by localized RT and CAR-T cells.	NCT03070327 (31)

In the clinic, patients with immunosuppressive tumors have few therapeutic options, have high morbidity, poor long-term survival, and comprise an urgent unmet clinical need. In this review, we will focus on the potential benefits and challenges for combining RT with CAR-T cells for the treatment of immunosuppressive tumors and provide insights into how to manipulate these two treatments to maximize clinical benefit.

## Current Challenges for CAR-T Cells That Can be Targeted by RT

Poor trafficking, tumor penetration, and persistence of CAR-T cells, as well as tumor antigen heterogeneity and immunosuppression, are all major barriers to the success of CAR-T therapy in solid tumors. In this section, we will outline some of the mechanisms by which RT can overcome such challenges.

### Insufficient Recruitment of CAR-T Cells

Efficient trafficking of CAR-T cells to solid tumors within peripheral tissue has proven to be a significant challenge. The tumor stroma comprises immunosuppressive cells, cancer-associated fibroblasts (CAFs), epithelium, endothelium, and extracellular matrix (ECM). Together these elements form a micro-environment that inhibits the anti-tumor immune response (33). CAFs are a heterogeneous cell population commonly present in most tumor stroma. CAF-mediated aberrant high-density ECM contributes to the exclusion of effector T cells by acting as barriers to immune cell infiltration (34). In addition, CAFs also exhibit pro-tumorigenic capacity by inhibiting effector T cells, polarizing macrophages towards an M2 phenotype, recruiting suppressor cells, and remodeling the ECM (35). Few studies have explored the impact of radiation on CAFs; however, it was reported that RT could alter the pro-tumorigenic status of the tumor stroma (20) and potentially increase CAR-T cell infiltration into the tumor (**Figure 1**. Step ③).

Tumor-associated hypoxia and dysregulated vasculature are further barriers to T cell access into tumor stroma. Immunosuppressive tumors, such as prostate cancer, present with a hypoxic environment that excludes the T cells from infiltration. These hypoxic zones in the TME also recruit and harbor suppressive cells such as MDSC (36). As one of the major influencers in the TME, tumor-associated vasculature also contributes to maintaining an immunosuppressive TME. Pro-angiogenic factors, such as vascular endothelial growth factor A (VEGF-A), can reduce T cell infiltration by disrupting their access to the tumor bed and also inhibit adhesion molecules on endothelium for immune cell extravasation, such as intercellular adhesion molecule (ICAM)-1 and vascular cell adhesion protein (VCAM)-1 (37). RT contributes to the improved normalization of tumor vasculature in the TME. In prostate cancer, fractionated RT altered the function of tumor vasculature to improve tumor reoxygenation (19). Real-time imaging analysis in a glioblastoma model revealed that RT also promoted CAR-T cell extravasation and local expansion leading to a synergistic benefit of the combination treatment (27). In addition, radiation was shown to induce increased expression of the integrins ICAM-1 and VCAM-1 on the endothelium of the vasculature in the TME (21, 22), these adhesion molecules are critical for transendothelial migration of CAR-T cells (**Figure 1**. Step ④).

The migratory activity of CAR-T cells is largely influenced by the CXCR3/CXCL9-11 chemokine receptor-chemokine axis (38). These T cell recruiting chemokines are produced by M1-like immune-stimulatory macrophages in response to the proinflammatory cytokine IFN-γ (39, 40). In addition, CD8^+^ T cell infiltration is also controlled by the CCR5/CCL5 axis (40). However, immunosuppressive tumors lack the pro-inflammatory environment and immune-stimulatory cells to produce the T cell recruiting chemokines. Some tumors also secrete chemokines that recruit suppressive cells, such as regulatory T cell (Treg)-recruiting CCL17/22 and myeloid-derived suppressor cell (MDSC)-recruiting CCL2 (41). As a result, the immunosuppressive tumors present a mismatched chemokine signature for T cell recruitment, leading to limited effector T cell homing and tumor infiltration (42). Radiation-induced inflammation in the TME can promote the recruitment of effector T cells by triggering macrophages, in an IFN-γ dependent manner, to produce increased levels of the CXCR3-reactive T cell chemoattractants CXCL9, CXCL10, and CXCL11 (17, 26) (**Figure 1**. Steps ①②). Radiation-induced activation of stimulator of interferon genes (STING) pathway was also shown to increase expression of CXCL10 in a mouse mammary carcinoma model refractory to immune checkpoint inhibitors (18).

### Immunosuppression in the TME

The TME comprises a complex network of tumor cells and the tumor stroma made up of endothelial cells, fibroblasts, extracellular matrix, and immune cells (43). Despite sufficient trafficking and expansion of CAR-T cells in TME, suppressor immune cell subsets and soluble mediators can render CAR-T cells exhausted and dysfunctional (44).

Tregs are key contributors to tumor-mediated immune suppression (45). In patients with recurrent glioblastoma, infiltration of Tregs in the TME dampened immune activity and promoted acquired resistance to CAR-T cell therapy (46). Other immunosuppressive subsets that have been shown to impact CAR-T cell function are MDSC and tumor-associated macrophages (TAM) (47, 48). MDSCs can generate a suppressive milieu of cytokines and metabolites such as IL-10, TGF-β, IL-1 receptor antagonist, nitric oxide, and arginase 1 to hinder the tumoricidal immune response (49–52). Neutralizing MDSCs by immunostimulatory agents, such as all-trans retinoic acid, preserved CAR-T cell proliferation and cytotoxic function, and resulted in reduced tumor burden in CAR-T treated mice in an osteosarcoma model (53). Similarly, TAM can be co-opted by tumor cells and polarized to an anti-inflammatory M2-like phenotype capable of hindering T cell responses by the production of inhibitory mediators (i.e., TGF-β, indoleamine 2,3-dioxygenase IDO) and expression of PD-L1 (54, 55). Direct depletion of TAM has proven ineffective in promoting ACT, however, re-wiring of TAM to a pro-inflammatory phenotype by anti-CD40 agonist or blocking AIM2 inflammasome can improve the performance of immunotherapies including CAR-T cells (56, 57).

Cytokines and metabolic factors can also contribute to tumor progression and the loss of immune surveillance. The accumulation of lactate and adenosine, by-products of abnormal cellular metabolism in the TME, favors the infiltration and expansion of suppressive TAM and MDSC and dampens the activity of T cells (58–61). Enrichment of the inhibitory cytokine TGF-β has also been documented in many cancers and exerts profound immunomodulatory properties to attenuate the cytotoxic potential of T cells and accelerate T cell dysfunction (62, 63). TGF-β can also polarize myeloid cells and B cells towards an immunosuppressive phenotype (64, 65). As such, TGF-β co-opts various immunosuppressive cells to indirectly counteract immune activation in the TME. Neutralizing the TGF-β signal in the TME has been shown to unleash potent T cell responses, thereby rendering tumors susceptible to immunotherapy, including CAR-T cell treatment (66, 67).

Taken together, depleting Tregs and MDSC, reprogramming the TAM, and blocking the associated soluble mediators can be crucial to rescuing anti-cancer immune activity. In this regard, RT has been broadly investigated for its TME altering capacity (16, 32). This rationale is further supported by the notion that irradiation can alter the phenotype of immunosuppressive cells in the TME (**Figure 1**. Step ⑤). RT has been shown to enrich the TME for M1-like macrophages and reduce the frequency of immunosuppressive M2-like macrophages and MDSC (23, 24, 68, 69) (**Figure 1**. Step ⑥). Reduction of MDSC in the peripheral blood of patients post conventionally fractionated RT (<2 Gy/fraction) has also been reported (70). Similarly, hypofractionated RT (>2 Gy/fraction), has also been shown to reduce the influx of MDSC in TME by downregulating the expression of VEGF (25). Taken together, RT can boost immune activation by altering the immunosuppression status of the TME to enhance CAR-T cell efficacy.

Aside from its direct ability to debulk tumors, RT can also engage host immune defenses by causing immunogenic cell death (ICD) (71). ICD is associated with the release of danger-associated molecular patterns (DAMP) and increased expression of neoantigens that can help facilitate the recruitment and activation of dendritic cells and subsequent priming of T cell responses with an expanded TCR repertoire (72, 73). Radiation-induced activation of the STING pathway induces expression of Type I IFNs and TNF, leading to an inflammatory microenvironment, which facilitates T cell activity (22, 74) (**Figure 1**. Step ⑦). Of note, the STING response induced by a single fraction of high-dose radiation is distinct from that when the total dose is fractionated into a series of smaller doses. Demaria and colleagues demonstrated that fractionated radiation schedules of less than 8 Gy/fraction activated the STING pathway and release of Type I IFN, permitting the induction of abscopal response when delivered in combination with ICB therapy. In contrast, a single fraction of 20 Gy RT increased the expression of TREX1 within tumor cells, preventing STING activation and its ability to augment the systemic anti-tumor activity of ICB therapy (18).

### The Effect of RT on CAR-T Cell Function

In addition to the effect of RT on the TME and T cell trafficking, radiation can also promote CAR-T cell function. Radiation can induce tumor cell stress ligands and it is an alternate mechanism through which RT may increase tumor-cell susceptibility to CAR-T cell-mediated killing (**Figure 1**. Step ⑧). DeSelm et al. showed that a single fraction of 2 Gy could augment TRAIL-mediated cytolysis by anti-sialyl Lewis-A CAR-T cells, leading to attenuated tumor growth in mice bearing heterogeneous pancreatic tumors (28). A similar effect was also observed when radiation enhanced the IFN-γ production of CAR-T cells in a glioblastoma mouse model (29). In addition, CAR-T cell expansion was correlated with RT in a patient with relapsed diffuse large B cell lymphoma (**Figure 1**. Step ⑨). The CAR-T cell transgene copies dropped initially post-infusion but increased dramatically after RT and persisted for more than 120 days, leading to a complete remission post combination treatment (30).

Radiation may also induce the expression of stress ligands that can be targeted by CAR-T cells. NKG2D CAR-T cells combined with local intracranial RT significantly reduced tumor burden and prolonged survival, which can be attributed to the upregulation of NKG2DL, such as RAE-1 and MULT-1 post-irradiation (29).

In addition to the local effects of RT, induction of systemic anti-tumor immune responses that control tumor growth outside the irradiation volume (known as the abscopal effect), was also reported (75). In a case study of a BCMA CAR-T cell clinical trial, combination with RT showed complete radiographic resolution including the innumerable sites outside the radiation site with no relapse in 9 months post-treatment (31).

More evidence is emerging that certain dose/fractions of RT can, directly and indirectly, affect the CAR-T efficacy against tumor cells, although further detailed mechanisms to explain the benefits of RT on CAR-T cells are still not clear and should be investigated further.

## Challenges for the Combination Therapy of RT and CAR-T Cells

Although RT and CAR-T therapy have shown therapeutic efficacy in treating some tumors, further consideration is needed when designing the combination therapy to gain the maximum clinical benefit.

For many cancer patients, RT is given post CAR-T cell infusion so the adoptive transferred cells are potentially vulnerable to radiation-induced induce apoptosis (76), however, *in vivo* data relating to this point remains scarce. Notably, antigen-experienced T cells, such as memory CD8^+^ T cells and tissue-resident memory T cells have been reported to be more resistant to radiation-induced apoptosis compared to naïve T cells (77, 78). Thus, the ex vivo culturing processes necessary to generate CAR T cells may aid in strengthening their resilience to direct exposure to external beam RT. In a clinical trial, low dose RT (2 Gy/fraction) was found to induce *in vivo* expansion of CAR-T cells (30). Based on our current knowledge of the immunological effects of RT we would expect that low dose RT (<2 - 4Gy) to be a better complement to CAR-T therapy. Low-dose RT is likely to be less impactful on the viability of the immune compartment and has reported positive impacts on TAM and the vasculature as discussed above.

Besides reprogramming the TAM and MDSC in the TME to a proinflammatory phenotype, RT can also induce the wound healing response and the induction of highly suppressive Treg responses (79). Interestingly, the rate and amplitude of accumulation of Tregs within irradiated tumors were dictated by dose per fraction rather than total dose. In this study, a single fraction of 20Gy generated a more aggressive Treg response compared to a fractionated dose of 9 x 4 Gy, despite sharing the same biological effector dose of 45Gy (79). Several preclinical studies have demonstrated that the targeted depletion of Tregs is required to induce a durable response to RT and support the anti-cancer actions of immune checkpoint blockade therapy (80, 81). Thus, a more precise dose/fraction should be considered, and additional Treg-targeted approaches may be needed to overcome potential immune suppression and acquired resistance. In this regard, other combination treatment strategies could be considered, such as chemotherapy and monoclonal antibodies. Docetaxel was reported to specifically deplete activated Tregs with more IFN-γ and less TGF-β, but not resting Tregs in non-small cell lung cancer (82). In pancreatic ductal adenocarcinoma, low-dose gemcitabine also induced Treg depletion (83). Another approach is to use the monoclonal antibodies (mAbs) targeting the key molecules on Treg cells, such as CTLA-4 (Cytotoxic T lymphocyte-associated antigen-4), CD73, and TIGIT (T cell immunoreceptor with Ig and ITIM domains). Some of these mAbs have been approved for treating cancers, such as Ipilimumab (anti-CTLA-4 mAb), and others are being investigated in clinical trials as reviewed elsewhere (84–86). Similarly, upregulation of inhibitory signaling molecules, such as PD-L1, have been observed on tumor cells post-radiation (87–89). Thus, adding immune checkpoint blockade into the combination therapy may resolve the negative effects of RT and promote the efficacy of CAR-T cells, although the safety and synergistic benefit remain to be evaluated in the clinic.

ATP released from irradiated tumor cells can be converted to adenosine by the ectoenzymes CD39 and CD73, which is another potential barrier for CAR-T cell function (90). Elevated adenosine impedes the anti-tumor response of effector T cells through their surface A2a adenosine receptors (A2aRs) (91–93). Blocking adenosine signaling has been shown to elicit a more potent T-cell response in combination therapy (94, 95). Besides therapeutic inhibitors, this strategy can also be achieved by directly modifying the CAR-T cells. The modification includes depleting the A2aRs in CAR-T cells by CRISPR-Cas9 editing and engineering CAR-T cells to carry antagonist nanoparticles. Both approaches showed increased efficacy with radiation in CD73/CD39 induced adenosine-enriched tumors (96, 97), but not in tumors with weak CD73/CD39 expression, such as melanoma (98). Therefore, for radiation-induced adenosine-rich tumors, inhibitors of the adenosine pathway or the engineering of CAR-T cells to resist adenosine-induced suppression may prove beneficial in the context of RT.

Radiation-induced enrichment of TGF-β in the TME is another critical barrier to the effective partnership of RT and CAR-T cells (26, 79). The wound healing process and DNA repair programs post-radiation treatment trigger TGF-β signaling in the TME, resulting in epithelial-mesenchymal transition, tissue fibrosis, and the induction of a broad spectrum of immunosuppressive effects on infiltrating immune cells including CAR-T cells (99, 100). To overcome this barrier, CAR-T cells can be modified to become resistant to TGF-β mediated immunosuppression. For example, Kloss et al. engineered a dominant-negative TGF-β receptor (dnTGF-βRII) capable of binding TGF-β without directly triggering a T-cell inhibitory signaling event. This approach proved effective in promoting superior anti-tumor efficacy compared to the parental CAR-T cells in a metastatic prostate cancer model (101). The TGF-β on CAR-T cells can also be blocked by knocking out the endogenous TGF-β type II receptor (102). However, given the homeostatic function of TGF-β signaling in lymphoid tissues, constitutive TGF-β blockade may result in off-target toxicity. Indeed, following treatment with dnTGFβRII-PSMA-CAR-T cells, a subset of patients developed severe cytokine release syndrome and immune effector cell-associated neurotoxicity syndrome (ICANS) (103). Based on these findings, efforts are ongoing to refine this approach. Developing a CAR construct to selectively capture and neutralize TGF-β only within the TME may indeed prove to be efficacious and safe.

## Conclusion

Combination treatment of RT and CAR-T cells has significant therapeutic potential. Although it is a promising option for patients with immunosuppressive tumors, further considerations on dose/fraction, treatment schedule, immune context, and tumor type should be considered when designing the treatment, and more mechanistic studies are still needed to understand how these therapies will best work in combination. Our increased understanding of the immunomodulatory effects of RT together with the incredible advances being made in the CAR-T cell field, especially with promising molecular engineering of novel CAR constructs, will facilitate the successful implementation of this combination strategy in the clinic.

## Author Contributions

Writing-original draft preparation: VQ, NH, and JZ. Writing-review and editing: NH, CD’S, PN, and JZ. Supervision: PN. All authors contributed to the article and approved the submitted version.

## Conflict of Interest

The authors declare that the research was conducted in the absence of any commercial or financial relationships that could be construed as a potential conflict of interest.

## Publisher’s Note

All claims expressed in this article are solely those of the authors and do not necessarily represent those of their affiliated organizations, or those of the publisher, the editors and the reviewers. Any product that may be evaluated in this article, or claim that may be made by its manufacturer, is not guaranteed or endorsed by the publisher.
